# Whole genome analysis of two sympatric human *Mansonella*: *Mansonella perstans* and *Mansonella* sp “DEUX”

**DOI:** 10.3389/fcimb.2023.1159814

**Published:** 2023-04-14

**Authors:** Miriam Rodi, Caspar Gross, Thaisa Lucas Sandri, Lilith Berner, Marina Marcet-Houben, Ersoy Kocak, Michaela Pogoda, Nicolas Casadei, Carsten Köhler, Andrea Kreidenweiss, Selidji Todagbe Agnandji, Toni Gabaldón, Stephan Ossowski, Jana Held

**Affiliations:** ^1^ Institute of Tropical Medicine, University of Tübingen, Tübingen, Germany; ^2^ Institute of Medical Genetics and Applied Genomics, University of Tübingen, Tübingen, Germany; ^3^ Laboratory of Molecular Immunopathology, Department of Clinical Pathology, Federal University of Paraná, Curitiba, Brazil; ^4^ Life Science Department, Barcelona Supercomputing Centre (BSC-CNS), Barcelona, Spain; ^5^ Mechanisms and Defense, Institute for Research in Biomedicine (IRB Barcelona), The Barcelona Institute of Science and Technology, Barcelona, Spain; ^6^ NGS Competence Center Tübingen (NCCT), Tübingen, Germany; ^7^ German Center for Infection Research (DZIF), partner site Tübingen, Tübingen, Germany; ^8^ Centre de Recherches Médicales de Lambaréné (CERMEL), Lambaréné, Gabon; ^9^ Catalan Institution for Research and Advanced Studies (ICREA), Barcelona, Spain; ^10^ Centro de Investigación Biomédica En Red de Enfermedades Infecciosas (CIBERINFEC), Barcelona, Spain

**Keywords:** *Mansonella* sp “DEUX”, *Mansonella perstans*, whole genome sequencing, phylogeny, *Wolbachia*

## Abstract

**Introduction:**

*Mansonella* species are filarial parasites that infect humans worldwide. Although these infections are common, knowledge of the pathology and diversity of the causative species is limited. Furthermore, the lack of sequencing data for *Mansonella* species, shows that their research is neglected. Apart from Mansonella perstans, a potential new species called *Mansonella* sp “DEUX” has been identified in Gabon, which is prevalent at high frequencies. We aimed to further determine if *Mansonella* sp “DEUX” is a genotype of M. *perstans*, or if these are two sympatric species.

**Methods:**

We screened individuals in the area of Fougamou, Gabon for Mansonella mono-infections and generated de novo assemblies from the respective samples. For evolutionary analysis, a phylogenetic tree was reconstructed, and the differences and divergence times are presented. In addition, mitogenomes were generated and phylogenies based on 12S rDNA and cox1 were created.

**Results:**

We successfully generated whole genomes for M. perstans and *Mansonella* sp “DEUX”. Phylogenetic analysis based on annotated protein sequences, support the hypothesis of two distinct species. The inferred evolutionary analysis suggested, that M. perstans and *Mansonella* sp “DEUX” separated around 778,000 years ago. Analysis based on mitochondrial marker genes support our hypothesis of two sympatric human Mansonella species.

**Discussion:**

The results presented indicate that *Mansonella* sp “DEUX” is a new *Mansonella* species. These findings reflect the neglect of this research topic. And the availability of whole genome data will allow further investigations of these species

## 1 Introduction

Infections with nematodes of the genus *Mansonella* are among the most neglected diseases, even though these parasites belong to the most widespread human filaria species. It has been estimated that more than 100 million people are infected with *Mansonella* species in Africa only and around 600 million people are at risk of infection (Simonsen et al., 2011). Three species are known to be prevalent in humans, *Mansonella perstans*, *M. ozzardi*, and *M. streptocerca* with varying geographic distribution. While *M. perstans* is prevalent in most parts of Sub-Saharan Africa and some regions in South and Central America, *M. streptocerca* is restricted to the African continent and *M. ozzardi* to Latin America. In 2015 a potential new species provisionally called *Mansonella* sp “DEUX” was described in febrile children in a hospital-based study in Gabon (Mourembou et al., 2015). Further investigations showed that this was the most frequent filarial species, as during a cross-sectional study in rural Fougamou, Gabon, 35% of individuals carried this parasite (Sandri et al., 2021).

In addition to the nuclear and the mitochondrial genomes, most filarial species comprise an additional genome - the genome of their bacterial endosymbiont *Wolbachia*. These intracellular bacteria can be found in most filarial parasites (Ferri et al., 2011). For *M. perstans* the obligate presence of *Wolbachia* was under debate for a long time (Grobusch et al., 2003; Gehringer et al., 2014; Batsa Debrah et al., 2019). However, improved molecular diagnostics, as well as results from treatment studies demonstrating the efficacy of antibiotics, indicate that *M. perstans* contains these bacteria (Keiser et al., 2008; Coulibaly et al., 2009; Gehringer et al., 2014; Batsa Debrah et al., 2019). *Wolbachia* were also detected in individuals mono infected with *Mansonella* sp “DEUX”, confirming carriage of endosymbionts also by this new species/genotype (Sandri et al., 2021). As *Wolbachia* are usually transferred maternally and because of the known genetic variability of this endosymbiont among nematodes, *Wolbachia* genomes could give a further hint on the genetic relatedness of their host species (Bordenstein et al., 2009).

Until recently, no whole genome for any *Mansonella* species infecting humans was available, reflecting the neglect of these infections. Fortunately, this has been recognized and sequences of *M. perstans* and *M. ozzardi* have been generated in the last years (Crainey et al., 2018; Chung et al., 2020; Sinha et al., 2023). A recently published study from Tamarozzi et al. has confirmed the lack of consensus in the management of *Mansonella* infections (Tamarozzi et al., 2022). There are no clear treatment guidelines and also information on specific symptoms and burden of disease is not well described. To contribute to a better evolutionary understanding of *Mansonella* species, we generated and compared the whole genomes as well as the mitogenomes of *M. perstans* and *Mansonella* sp “DEUX” isolated from circulating microfilariae from infected individuals. We aimed to assess the differences between the two sympatric variants to identify if they are two separate species. In addition, we also obtained and analyzed the genomes of the *Wolbachia* symbionts.

## 2 Materials and methods

### 2.1 Ethical statement

The study protocol was approved by the Institutional Ethics Committee at the Centre de Recherches Médicales de Lambaréné, Gabon (CEI-CERMEL 002/2018). Adolescents were asked to give written assent, written informed consent was obtained from their legal guardian and of all adult individuals taking part in this study.

### 2.2 *Mansonella perstans* and *Mansonella* sp “DEUX” sample collection

Individuals ≥ 16 years living in the area of Fougamou, Gabon, with a confirmed *Mansonella* species microfilaria count ≥ 50 parasites/ml and no microscopical detectable *Loa loa* infection were invited to take part in the study. Diagnosis was based on the microscopic reading of two Giemsa-stained thick blood smears (2x 10 µl blood). 18 ml of venous blood was collected in EDTA tubes from participating individuals. The sample was diluted 1:2 with phosphate buffered saline (PBS; Merck) and filtered through a 5 µm filter paper (Whatman) with a syringe to enrich the microfilariae. Filtration was repeated with 30 ml of PBS to remove remaining blood cells. Subsequently the Whatman filter was removed, the sticking microfilariae were washed off with 10 ml PBS, and the mixture was centrifuged (10 minutes, 1800 rpm) to remove the supernatant. The pellet was transferred to a cryotube with RNAlater and frozen at -80°C.

### 2.3 DNA extraction and *Mansonella* species confirmation

DNA was extracted from a 25 µl pellet using NEB Monarch Genomic Purification Kit (NEB). Sample lysis was performed according to the animal tissue protocol provided by the manufacturer. Incubation was increased to 3 h at 56°C to improve the yield. DNA was eluted with 50 µl elution buffer. *Loa loa* infection as well as coinfection with either *M. perstans* or *Mansonella* sp “DEUX” based on the internal transcribed spacer region (ITS1 region) was excluded by qPCR according to previously published protocols (Sandri et al., 2021). These procedures resulted in three eligible samples, named mperst1, mdeux2 and mdeux3.

### 2.4 Genome sequencing, *de novo* assemblies and genome annotation

#### 2.4.1 Library preparation for whole genome sequencing

Genomic DNA was quantified using Qubit dsDNA HS assay kit with a Qubit fluorometer (ThermoFisher). DNA library preparation was performed using the Nextera DNA Flex kit (Illumina) following the manufacturers instruction. Briefly, 4 to 20 ng of genomic DNA was diluted in a total volume of 30 µl of nuclease-free water and fragmented at 55°C for 15 minutes using tagmentation. The enzymatic reaction was stopped, and the adapter-tagged DNA was purified using magnetic-bead clean-up. The resulting DNA was amplified using 8 to 12 cycles of PCR, allowing the required sequencing adapters to anneal to the DNA. The resulting libraries were purified using a double-sided beads purification. Library molarity was determined by measuring the library size (approximately 600 bp) using the Fragment Analyzer with the High NGS Fragment 1-6000bp assay (Agilent) and the library concentration (approximately 10 ng/µl) using the Infinite 200Pro (Tecan) and the Quant-iT HS Assay Kit (Thermo Fisher Scientific). The libraries were denaturated, diluted to 162 pM and sequenced as paired-end 250 bp reads on an Illumina NovaSeq6000 (Illumina, San Diego, CA, USA) with a sequencing depth of approximately 200 million clusters per sample.

#### 2.4.2 *De novo* assembly and genome annotation

The first step in the *de novo* assembly of the *Mansonella* species genomes was to remove the reads from the human host from all generated reads. Therefore, Kraken2 (Wood et al., 2019) was used to classify the reads taxonomically since unmapped reads may also indicate the presence of *Wolbachia* sequences. The reads reported as unclassified by Kraken were assumed to be *Mansonella* species reads and used for the *de novo* assembly. Information about the pre-built Kraken database used can be found in the **Supplementary File 1**. The assembly was performed using the SPAdes *De Novo* Assembler (Bankevich et al., 2012) without error correction, using the default parameters. The quality of the scaffolds assembled by SPAdes were assessed using the genome assembly evaluation tool QUAST (Gurevich et al., 2013) and the genome completeness was assessed using BUSCO (Simao et al., 2015). Also, the N50 values and coverage values of the scaffolds were calculated in this way. BLAST (Altschul et al., 1990) was used to investigate the similarity of the potential *Mansonella* species scaffolds to closely related species. If at least one of the 39 listed species (**Supplementary Table 1**) occurred in the top 10 BLAST hits of a scaffold, the scaffold was counted as a fragment of the *Mansonella* species genome. Estimated genome length was calculated by summing the lengths of these filtered scaffolds. Intron hints for the gene annotation were generated using the protein sequences of *B. malayi* with the freely available sequence alignment program, Exonerate (Slater and Birney, 2005). Subsequently, AUGUSTUS (Stanke and Morgenstern, 2005) gene prediction tool was used to predict the genes on the scaffolds using the hints and to annotate the predicted genes **Supplementary File 1**. The predicted genes were aligned to the NCBI protein sequence database using DIAMOND (Buchfink et al., 2021) to find similar genes.

### 2.5 Evolutionary analysis of *Mansonella* species genomes

#### 2.5.1 Phylome reconstruction

The evolutionary analysis was performed using genome assemblies from 10 nematodes, that were downloaded from NCBI and Uniprot (**Supplementary Table 2**), in addition to mperst1 and mdeux3. Genome completeness was assessed using BUSCO v4.0.2 (**Supplementary Table 2**) (Simao et al., 2015). The phylome of *Mansonella* sp “DEUX” was reconstructed from mdeux3 using a set of 11 additional Nematode proteomes, including mperst1. The genomes of *Brugia timori* and *Onchocerca flexuosa* were not used in the phylome reconstruction due to their poor completeness, as well as mdeux2. An automated pipeline was used that applies the same process to reconstruct gene trees as one would do manually (Fuentes et al., 2022). First the proteome database was reconstructed using the 12 species and formatting the codes to phylomeDB format. Then a blastp search was performed between each gene in the genome of *Mansonella* sp “DEUX” and this proteome database. Blast results were filtered using an e-value threshold of 1e-05 and an overlap threshold of 50%. The number of hits was limited to the 150 best hits for each protein. Next, six different multiple sequence alignments were reconstructed using three programs [Muscle v3.8.1551 (Edgar, 2004), mafft v7.407 (Katoh et al., 2005) and kalign v2.04 (Lassmann and Sonnhammer, 2005)] and aligning the sequences in forward and in reverse direction. From this group of alignments, a consensus alignment was obtained using M-coffee from the T-coffee package v12.0 (Wallace et al., 2006). Alignments were then trimmed using trimAl v1.4.rev15 (consistency-score cut-off 0.1667, gap-score cut-off 0.9) (Capella-Gutierrez et al., 2009). IQTREE v1.6.9 (Nguyen et al., 2015) was used to reconstruct a maximum likelihood phylogenetic tree. Model selection was limited to 5 models (DCmut, JTTDCMut, LG, WAG, VT) with freerate categories set to vary between 4 and 10. The best model according to the BIC criterion was used. 1000 rapid bootstraps were calculated. All trees and alignments were stored in phylomedb (Fuentes et al., 2022) with phylomeID 97 (http://phylomedb.org).

#### 2.5.2 Species tree reconstruction

A species tree was reconstructed using a supertree approach as implemented in duptree v1.48 (Wehe et al., 2008) using all the trees reconstructed in the phylome as input. A second species tree was reconstructed using a gene concatenation approach, adding the three genome assemblies from the species that were not initially included in the phylome reconstruction process (*B. timori* and *O. flexuosa*, as well as the second *Mansonella* sp “DEUX” sample mdeux2). In order to do that, genes in the phylome were selected that were found in single copy in all species. 1068 such genes were selected, and a blast search was performed between these genes and the proteomes of the three newly added assemblies. Only those genes were kept that had only one hit in each of the three new assemblies, reducing the set to 562 genes. The alignment was redone for each selected protein family, adding the three new sequences, and using the same pipeline applied during phylome reconstruction. These alignments were subsequently concatenated into a single multiple sequence alignment which was trimmed using trimAl v1.4.rev15 (Capella-Gutierrez et al., 2009), producing an alignment of 260.126 amino acid positions. IQTREE v1.6.9 (Nguyen et al., 2015) was used to reconstruct the species tree using the model selection option without restrictions. The best model according to BIC was JTT+F+R4.

#### 2.5.3 Nucleotide divergences and SNP calling

Nucleotide divergence was calculated by performing a best bidirectional hit analysis between all pairs of *Brugia*, *Onchocerca* and *Mansonella* species. For pairs of orthologs, the percent of identity was calculated by aligning 100 random pairs of nucleotide sequences using Muscle (Edgar, 2004) and using trimAl (Capella-Gutierrez et al., 2009) to calculate the pairwise identity without considering gaps.

#### 2.5.4 Divergence time calculation

Fossil records for Nematodes were obtained from Paleobiology Database (https://paleobiodb.org/). Four fossils fell within the species tree used in this paper: *Cascofilaria baltica* (33.9 - 38.0 MyA) and *Cascofilaria dominicana* (13.82 - 20.44 MyA) which belong to the Filariidae family, as well as *Ascarites gerus* (129.4 - 122.46 MyA) and *Ascarites rufferi* (237 - 242 MyA), which belong to the Ascarididae family. SortaDate (Smith et al., 2018) was used to select the 20 best genes to use for dating based on their clock-likeness, tree length and topological similarity to the species tree. Trees were used from the phylome, despite having three genome assemblies not included. Alignments were then concatenated, a species tree was reconstructed as detailed above, and phylobayes was used to calculate divergence times using the two calibration points based on the fossil records. The rooting point was set to have happened at least 242 MyA without detailing an upper limit. The second calibration point was set at the base of Filarioidea and was set to have happened between 13 and 38 MyA. Phylobayes 4.1 (Lartillot et al., 2009) was run on the species tree and with those calibration points using two chains, the CAT model and the Birth and Death model. It was run until both chains converged as seen with tracecomp from the Phylobayes package. Readdiv was run to obtain the chronogram using a burn-in of 10.000 and a subsampling frequency of 10.

### 2.6 Extraction of mitogenome assemblies

The mitogenomes were extracted from the whole genome assembly of the individual samples. Using the visual tool Bandage (Wick et al., 2015) the *M. perstans* mitogenome was blasted against the assembly graph to identify contigs with mitochondrial genomic sequences. In case of multiple matching contigs these were linked based on coverage and sequence similarity to extract one or more complete mitogenomes from the assembly.

#### 2.6.1 Phylogenetic tree reconstruction based on mitogenomic data

The analysis follows the companion repository from Chung et al. (2020). First, a multiple sequence alignment including the mitogenomes of mperst1, mdeux2 and mdeux3 as well as *Mansonella* related species from NCBI (**Supplementary Table 3**) was created using CLUSTAL OMEGA v.1.2.3. Then the phylogeny was calculated using IQTREE v2.1.4 (Nguyen et al., 2015) and visualized using iTOL (Letunic and Bork, 2021). For the 12S rDNA phylogenies, rRNA sequences were extracted from the genome assemblies using BARRNAP v0.9 Seemann, T (2013) and then combined with published 12S rDNA sequences from other *Mansonella* species. 12S rDNA and *cox1* phylogenies were created using the same methods described above for the metagenome data.

## 3 Results

### 3.1 *De novo* assembly and gene annotation

Genome assemblies were generated from *Mansonella* microfilaria isolated from three individuals living in the area of Fougamou, Gabon. They presented with a qPCR-confirmed mono-infections of either *M. perstans* (individual 1: mperst1) or *Mansonella* sp “DEUX” (individual 2: mdeux2 and individual 3: mdeux3) (**Table 1**). Reads for all three samples were obtained with a coverage of 7.4x (mperst1), 32.4x (mdeux2), and 65.5x (mdeux3), resulting in a total assembly size of 76.2 Mb, 80 Mb and 78.5 Mb, respectively (**Table 2** and **Supplementary Table 4**). Although the generated whole genome sizes are comparable, the N50 from mdeux3 is twice and three times the size of the other two assemblies respectively. This makes mdeux3 the most contiguous assembly generated thus far. To further verify the quality of the assemblies, the completeness of the data was quantified in terms of the expected gene content, using BUSCO (Benchmarking Single-Copy Orthologs) tool. For all three samples, more than 90% of conserved eukaryotic gene orthologs were identified. GC content as well as the number of predicted genes are comparable among all three samples.

**Table 1:** Infection status, parasitemia and demographic data of individuals for mperst1, mdeux2 and mdeux3.

Sample ID	Species based on ITS1 qPCR	Ct value	Microscopic count [mf per ml]	Participant sex	Participant age [years]	Participant living area
**mperst1**	*Mansonella perstans*	25	350	female	71	Nzong Bang, Lambaréné, Gabon
**mdeux2**	*Mansonella* sp “DEUX”	26	125	male	78	Nzemba, Lambaréné, Gabon
**mdeux3**	*Mansonella* sp “DEUX”	24	400	male	70	Moukabou, Lambaréné, Gabon

**Table 2:** Comparison of genome assemblies mperst1, mdeux2 and mdeux3 to *L. loa*. A more detailed comparison to other filarial species can be found in **Supplementary Table 4**.

	mperst1	mdeux2	mdeux3	*L. loa*
**Total length (Mb)**	76.2	80.0	78.5	91.4
**GC (%)**	30.3	30.3	30.3	31.0
**N50 (kb)**	45.5	101.8	173.8	174.4
**Complete BUSCO (%)**	94.7	94.4	96.0	95.4

The respective mitochondrial and *Wolbachia* genomes were assembled independently for all three samples. *Wolbachia* reads were sequenced and assembled from all three samples called *w*Mpe1 for mperst1, *w*Mde2 for mdeux2, and *w*Mde3 for mdeux3. The *Wolbachia* assemblies are of comparable low quality (**Supplementary Table 5**). As a comparison, *Wolbachia* assemblies from other hosts were included. Among them are *Wolbachia* from supergroup F, as well as from supergroup D. Overall the here generated *Wolbachia* assemblies show a slightly lower GC content and a comparably high number of small contigs. In general, the assemblies are shorter than those used for comparison, especially *w*Mpe1 is four times smaller than the reference *w*Mpe (JACZHU01.1). Due to the poor quality, further analyses are not presented here.

### 3.2 Evolutionary analysis indicates a divergence between *Mansonella perstans* and *Mansonella* sp “DEUX”

**Figure 1:**
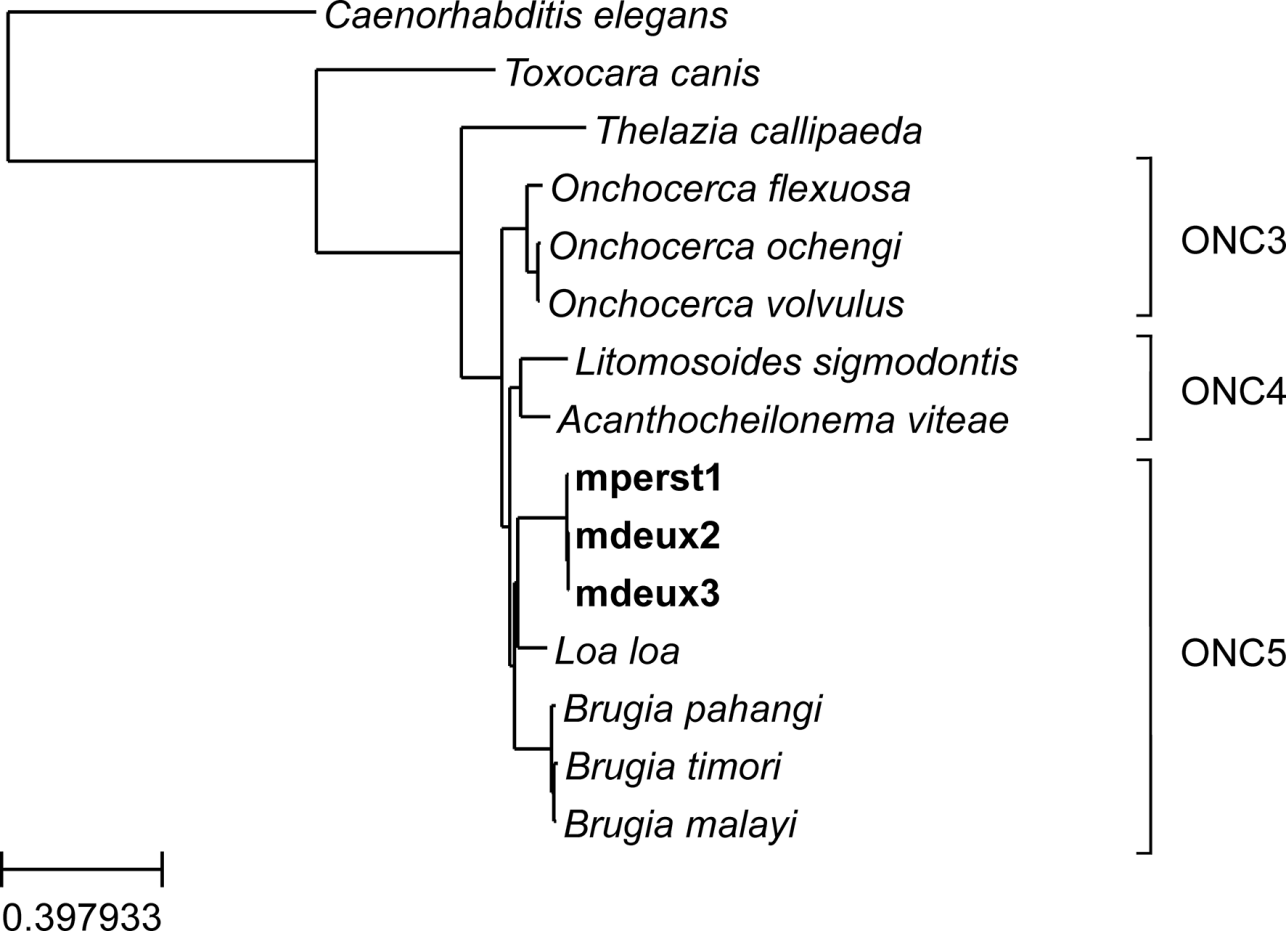
A phylogenetic tree reconstructed for *M. perstans* assembly mperst1 and the two *Mansonella* sp “DEUX” assemblies mdeux2 and mdeux3, including 12 other nematode species based on the concatenation of 562 protein alignments. *Onchocercidae* clades ONC3, ONC4 and ONC5 are shown next to the corresponding species.

To provide further information on the orthology and paralogy relationships across the genes of the nematode species, a phylome – i.e., a complete catalogue of gene evolutionary histories - was reconstructed for *M. perstans* from mperst1 and *Mansonella* sp “DEUX” from mdeux3, including 10 other sequenced nematode species. This allowed the selection of widespread genes that were present in single copy in the selected species. Subsequently, these genes were also identified in the second *Mansonella* sp “DEUX” sample mdeux2 as well as in two additional nematode species which were not included in the phylome analyses due to the fragmented nature of their assemblies (*Brugia timori* and *Onchocerca flexuosa*). This set of widespread genes was used to build a concatenated alignment and then a species tree was reconstructed (**Figure 1**). Using the inferred branch lengths from this tree, the phylogenetic distances within groups of species or strains from the same genus (*Mansonella, Brugia* and *Onchocerca*) were compared. As seen in **Figure 1**, branch lengths are very short for all three species groups, though the branches are shortest between the two *Mansonella* sp “DEUX” strains mdeux2 and mdeux3. The branch separating *Mansonella* sp “DEUX” and *M. perstans* is similar in length to the one separating *Onchocerca ochengi* and *O. volvulus* and the one separating *B. timori* and *B. malayi*, indicating that, at a genomic level, they have a similar genetic divergence. These findings are further confirmed by the calculations of nucleotide and amino acid diversity between the species in the three genera *Mansonella*, *Brugia* and *Onchocerca* (**Supplementary Table 6**). These analyses support the separation of *Mansonella* sp “DEUX” and *M. perstans* into two different species, as their genetic divergence is similar to that between established nematode species.

Our phylogenetic analysis supports the results of the multi-locus sequence typing analysis by Lefoulon et al. (2015), which suggests a division of *Onchocercidae* into distinct phylogenetic clades (ONC1-ONC5). **Figure 1** additionally shows the phylogenetic *Onchocercidae* clades ONC3, ONC4, and ONC5. Consistent with Lefoulon et al., all marked species are placed into the same clades. *O. flexuosa*, *O. ochengi*, and *O. volvulus* into clade ONC3, *Litomosoides sigmodontis* and *Acanthocheilonema vitae* into clade ONC4*. Loa loa, B. pahangi, B. timori, B. malayi* as well as the three *Mansonella* assemblies from mperst1, mdeux2 and mdeux3 are placed into clade ONC5.

**Figure 2:**
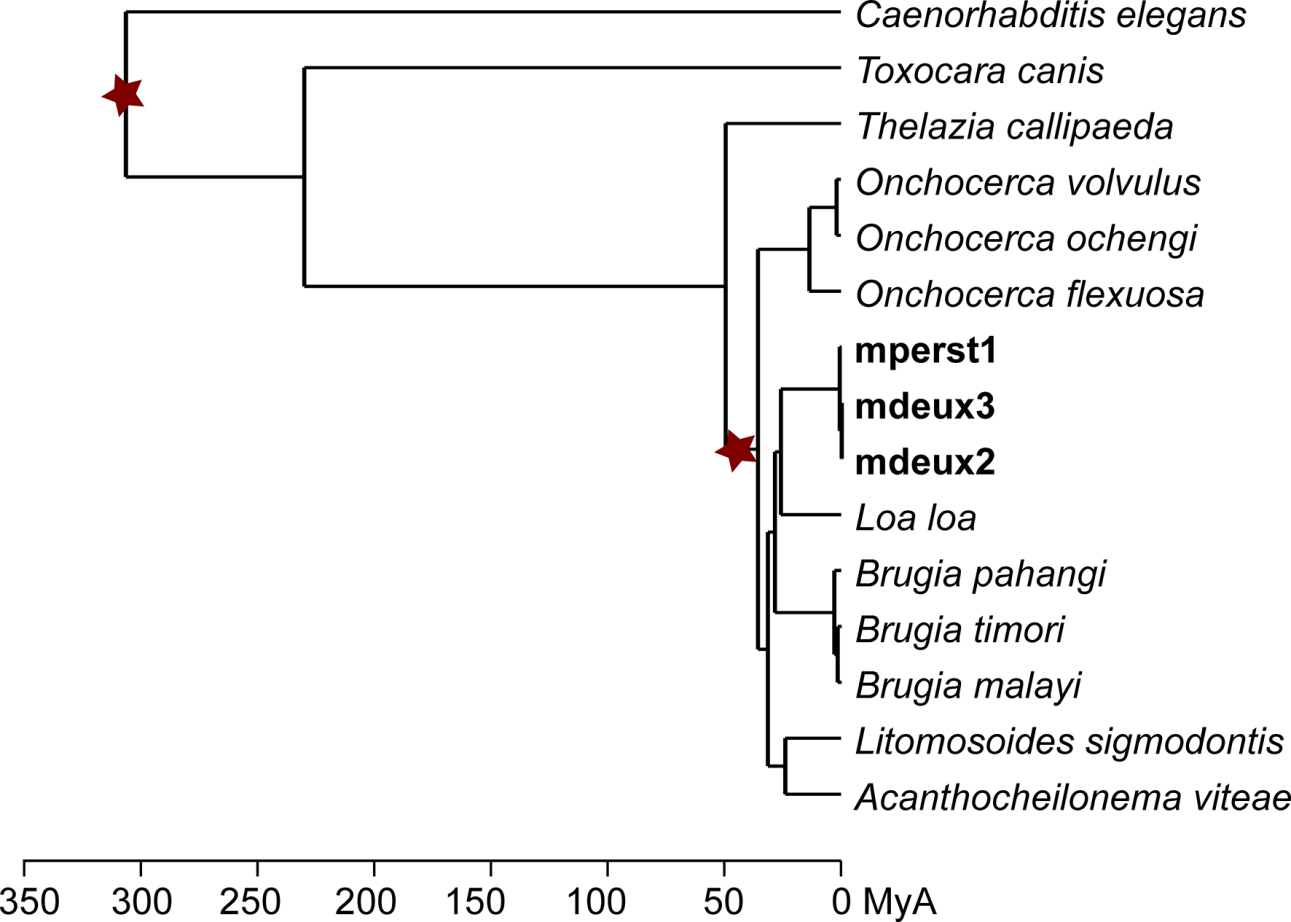
Evolutionary analysis of *Mansonella* sp “DEUX” and *M. perstans*. Chronogram detailing the divergence times of 15 nematode species. Stars indicate calibration points derived from recorded fossil evidence.

To calculate the divergence time of mperst1 from mdeux2 and mdeux3, a chronogram was generated (**Figure 2**). Two calibration points were set based on fossil records, one placed at the root of the tree and the other at the base of the Filarioidea super family. Using this calibration, the divergence time between *Mansonella* sp “DEUX” and *M. perstans* is estimated at 778,000 years. To better classify these time spans, we give the divergence time of the closest members of *Brugia* (1.6 MyA) and *Onchocerca* (1.8 MyA) for comparison. The divergence time of the two strains of *Mansonella* sp “DEUX” is 33,000 years.

### 3.3 Phylogeny based on mitochondrial sequences reveals three distinct *Mansonella* mitogenomes

Sequence information for filaria species is rare, however a mitochondrial genome of *M. ozzardi* and *M. perstans* has been published recently (Crainey et al., 2018; Chung et al., 2020). Therefore, a phylogenetic analysis based on the mitogenomes was performed to further understand the relationship between *M. perstans* and *Mansonella* sp “DEUX”.

**Figure 3:**
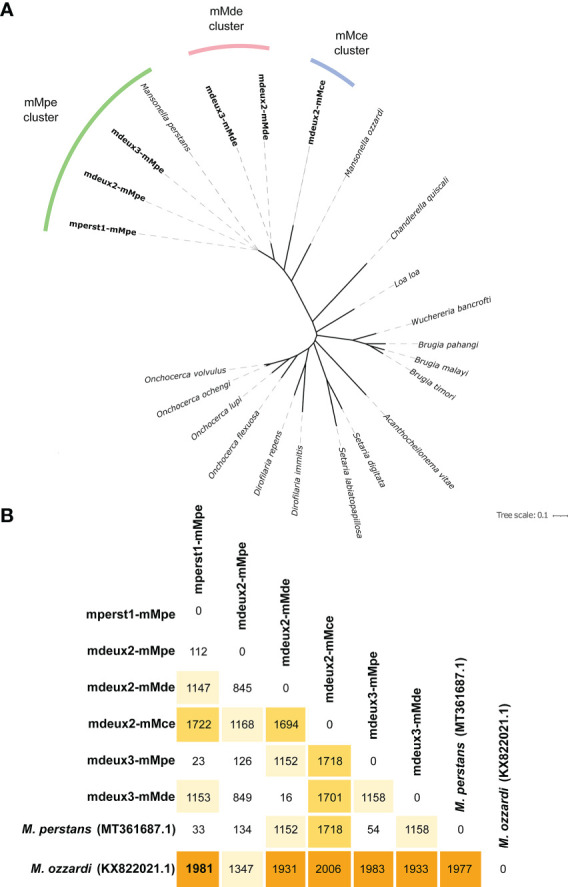
Mitogenome analysis. **(A)** Mitogenome phylogeny of different nematode species, including the newly generated mitogenomes from the three assemblies forming three *Mansonella* mitogenome clusters: Mitogenome of *M. perstans* mMpe marked in green, comprising of the mitogenomes from mperst1, and additionally two mitogenomes at low coverage of the individuals mdeux2 and mdeux3. *Mansonella* sp “DEUX” mMde marked in orange, with two mitogenomes of high coverage of mdeux2 and mdeux3. And the surprising third mitogenome of “*M. cermeli*” mMce with a single mitogenome of individual 2 (mdeux2). **(B)** SNP variant call between the three mitogenomes of each individual generated by us and the published mitogenomes of *M. perstans* (GenBank: MT361687) and *M. ozzardi* (GenBank: KX822021).

**Figure 3A** shows the generated phylogeny based on the mitogenomes of 17 nematode species, including the newly generated mitogenomes from the three assemblies. In total, three distinct mitogenome clusters from different species were identified from the assemblies. The first mitogenome cluster, designated as mMpe (mitogenome *M. perstans*), can be assigned to *M. perstans*, as it clusters with the published *M. perstans* mitogenome (GenBank: MT361687). It is found in mperst1 with a coverage of 40x. Unexpectedly, although identified as mono-infected with *Mansonella* sp “DEUX” *via* qPCR, assemblies of the individuals mdeux2 and mdeux3 were found to be mixed infected at low coverage with *M. perstans*. Both have in addition one mMpe mitogenome each, with lower coverage depths of 7x and 12x compared to the published *M. perstans* mitogenome, respectively. However, there were also two additional mitogenomes identified in each of these two assemblies that cluster together, both with a high coverage of 140x and 290x respectively. They show no similarity to any published mitogenomes and were therefore assigned to *Mansonella* sp “DEUX” and designated as mMde (mitogenome *Mansonella* sp “DEUX”). Surprisingly, a third mitogenome not clustering to the other mitogenomes or any other published sequence was identified in assembly mdeux2. Though it was sequenced at low coverage of 8x, it was assembled into a single contig, therefore confidence in the sequence is high. As the phylogeny suggests that this mitogenome originates from a separate species we provisionally call it “*Mansonella cermeli”* and the respective mitogenome mMce.

To compare the similarity of the generated mitogenomes, a SNP variant call was performed, including the reference mitogenomes of *M. perstans* (MT361687) and *M. ozzardi* (KX822021) (**Figure 3B**). The highest number of SNPs is found between each mitogenome and *M. ozzardi*. Whereas the lowest number of SNPs is found between the mitogenomes within one cluster as well as the mitogenomes from the cluster mMpe and *M. perstans*. Both samples from the mMde cluster (mdeux2-mMde and mdeux3-mMde) as well as the mMce cluster have a high number of SNPs compared to the mMpe cluster and the two references from *M. perstans* and *M. ozzardi*. This further supports the findings from the phylogenetic tree. Overall, genome size and GC content from all generated clusters are comparable to the published mitogenomes from *M. perstans* and *M. ozzardi* for all mitogenomes that were generated (**Table 3**). Both *Mansonella* sp “DEUX” mitogenomes mdeux2-mMde and mdeux3-mMde have a high coverage and are very similar to each other, showing a 99.8% pairwise identity. The same applies for the three *M. perstans* mitogenomes mperst1-mMpe, mdeux2-mMpe and mdeux3-mMpe. However, mdeux2_mMpe has a lower coverage and could not be completely assembled. The other two *M. perstans* mitogenomes though show a high similarity to the published *M. perstans* mitogenome. These data further support our hypothesis of two sympatric human *Mansonella* species.

**Table 3:** Comparison of different characteristics of the generated mitogenome clusters, including the published mitogenomes from *M. perstans* (MT361687) and *M. ozzardi* (KX822021).

Mitogenome	Length	GC (%)	Found in sample	Coverage
**mMpe**	13,616 bp	26.0	mperst1	40x
	8,978 bp	26.3	mdeux2	7x
	13,617 bp	26.0	mdeux3	12x
**mMde**	13,619 bp	25.5	mdeux2	140x
	13,621 bp	25.5	mdeux3	290x
**mMce**	12,613 bp	26.3	mdeux2	8x
***M. perstans* (MT361687)**	13,619 bp	25.9	NA	1,115x
***M. ozzardi* (KX822021)**	13,681 bp	25.7	NA	NA

NA, not applicable.

### 3.4 *Mansonella* sp “DEUX” infects humans as well as great apes

Parasites from the genus *Mansonella* are also known to infect non-human primates (NHPs). Only recently, filarial DNA extracted from NHPs faecal samples from Gabon and the neighbouring country Cameroon was used to investigate the *Onchocercidae* phylogeny based on two mitochondrial genes, cytochrome c oxidase subunit 1 (*cox1)* and 12S rDNA (Gaillard et al., 2020). These phylogenetic analyses showed that sequences obtained from chimpanzee cluster with samples from humans infected with *M. perstans*, concluding that chimpanzees can also be infected with *M. perstans*. In addition, they found NHPs to be infected with sequences of filaria that fall within the *Mansonella* genus, but cluster separately and are different from all published sequences.

**Figure 4:**
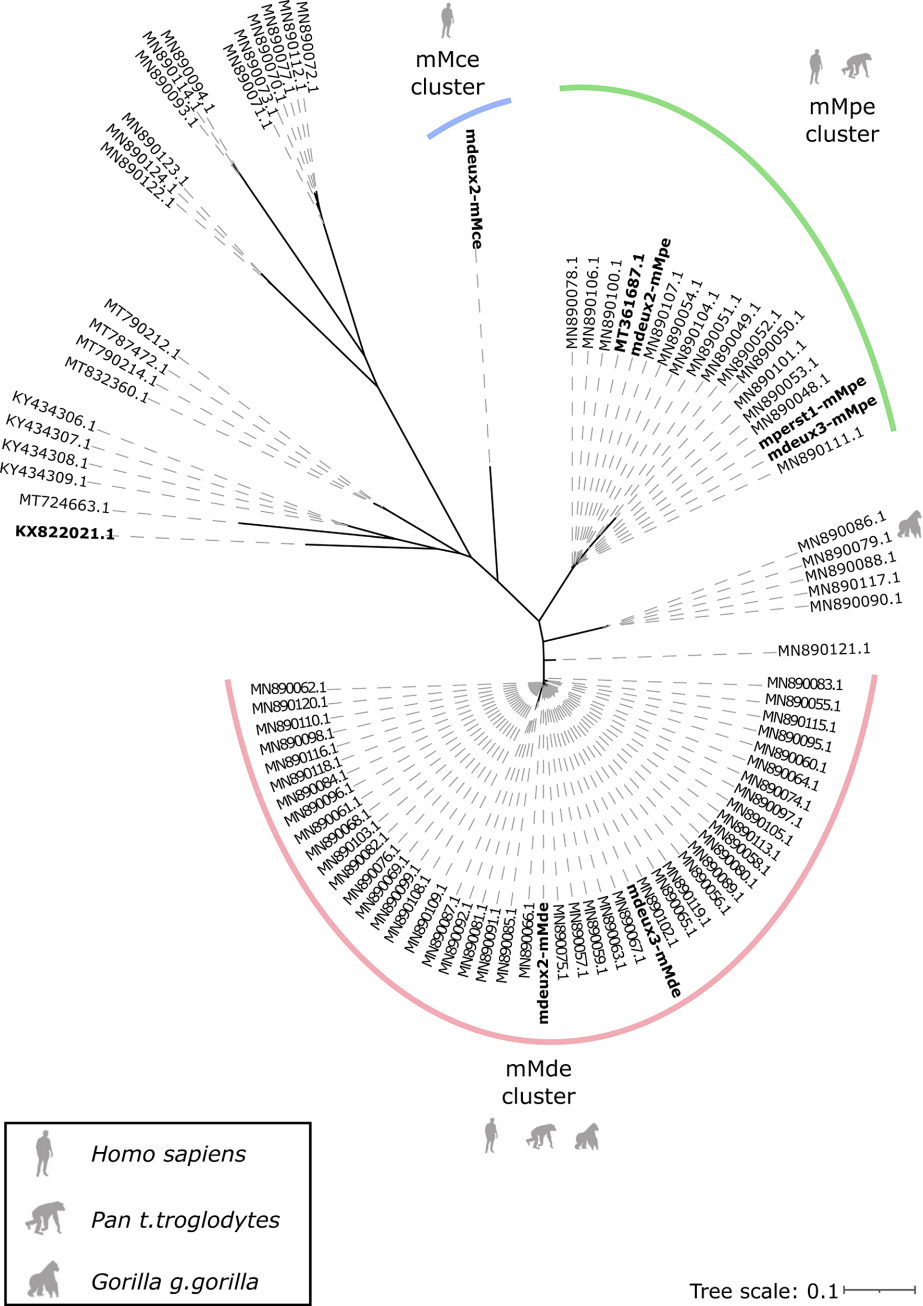
Phylogeny based on *cox1* gene sequences. The three mitogenome clusters mMpe, mMde and mMce are highlighted in green, red and blue respectively. The generated mitogenomes, as well as the published mitogenomes from *M. perstans* (GenBank: MT361687) and *M. ozzardi* (GenBank: KX822021) are highlighted in bold.

**Figure 5:**
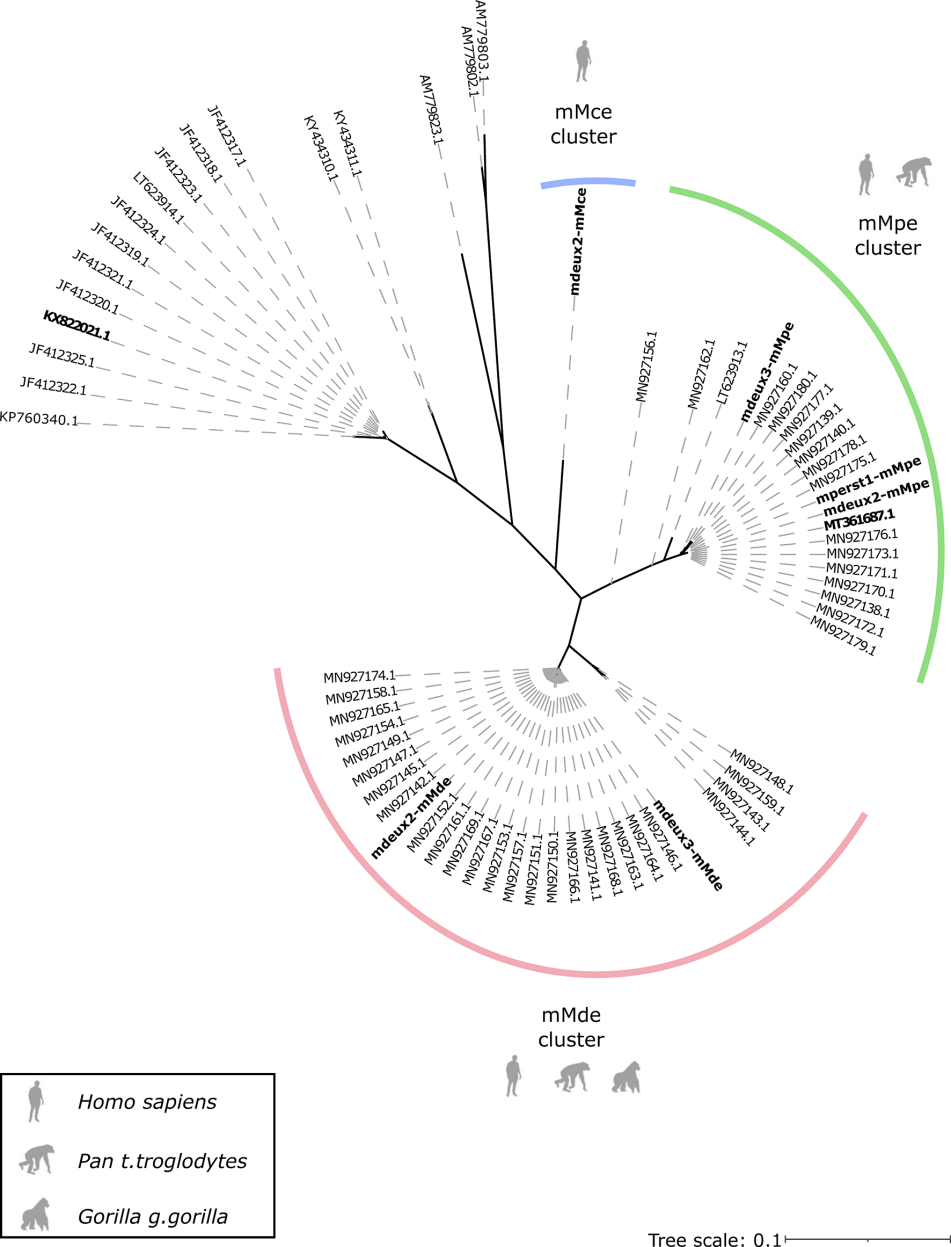
Phylogeny based on 12S rDNA gene sequences. The three mitogenome clusters mMpe, mMde and mMce are highlighted in green, red and blue respectively. The generated mitogenomes, as well as the published mitogenomes from *M. perstans* (GenBank: MT361687) and *M. ozzardi* (GenBank: KX822021) are highlighted in bold.

We extended the published phylogenetic trees from Gaillard et al. with the respective *cox1* (**Figure 4**) and 12S rDNA (**Figure 5**) sequences derived from the newly generated mitogenomes. As expected, the *cox1* sequences of three mitogenomes of the mMpe cluster, map to the published sequences by Gaillard et al., that cluster to *M. perstans*. The *cox1* sequences of the *Mansonella* sp “DEUX” mitogenomes (mMde) map to the unclassified *cox1* sequences of NHPs that could not be assigned to a *Mansonella* species by Gaillard et al. Therefore, it can be assumed that *Mansonella* sp “DEUX” infects humans and great apes. *Cox1* sequences of the mMce mitogenome cluster separately. Overall, we see the same five monophyletic groups, as depicted in Gaillard et al. The phylogenetic tree (**Figure 5**) based on 12S rDNA clusters corresponds very well to the clusters seen for *cox1.* We see separate clusters for the potential different species, as sequences from the *M. perstans* assemblies (mMpe assemblies: mperst1-mMpe, mdeux2-mMpe and mdeux3-mMpe) cluster to the published *M. perstans* sequences. This cluster can therefore be assigned to *M. perstans*. The 12S rDNA sequence from the mMce mitogenome forms a separate cluster. A third cluster is generated from two 12S rDNA sequences potentially originating from the *Mansonella* sp “DEUX” mitogenome assemblies mMde and additional 12S rDNA sequences generated from Gaillard et al., that were formerly grouped together with *M. perstans* sequences.

## 4 Discussion

Infections with *Mansonella* species have been neglected in all areas, this is also reflected by the fact, that until recently no whole genome of any of the *Mansonella* species was available. We generated whole genomes of *Mansonella perstans* and the related *Mansonella* sp “DEUX” from microfilaria of infected individuals from Gabon. All assemblies show a high level of quality, as is indicated by high coverage, N50 values and BUSCO scores. The whole genomes are comparable in size, number of predicted genes and GC content to other filarial species. Phylogenetic analysis of the whole genomes as well as the mitogenomes of these two *Mansonella* suggest that these are two distinct species. Evolutionary analysis of the whole genome comparisons supported these findings as they give estimates that these sympatric species have diverged 778,000 years ago.

However, we found sites of recombination between the whole genomes of *M. perstans* and *Mansonella* sp “DEUX”. This can be a true finding, indicating that these species are still able to cross in rare occasions. On the other hand, this can also be due to our whole genome assemblies. Based on mitogenomic data, we discovered that the *Mansonella* sp “DEUX” samples, which we originally assumed to be mono-infected, are in fact co-infected with *M. perstans* and/or an unknown potential third species “*M. cermeli”* at a low coverage. These co-infections have influenced our analysis, and it might be that they are responsible for the observed recombination regions that could lead to less accurate estimates. The relatively high divergence time of 33,000 years between the two *Mansonella* sp “DEUX” assemblies could also be explained by the influence of the co-infections in those samples. However, the overall results provide a good starting point for further research. With the collection of more *Mansonella* samples from different regions in the future, the genetic differences between samples of the same species can be estimated and can be included in the equations, and the divergence times can be calculated more accurately.

The first previously sequenced and published mitogenome of *M. perstans* from a sample from Cameroon (Chung et al., 2020) clusters closely to our *M. perstans* mitogenome and its inclusion in the phylogenetic analysis additionally supports the finding of two distinct species. Since sequence data on *Mansonella* species from different regions are limited, we additionally investigated the genes for which the most sequence data are available: *cox1* and 12S rDNA. Based on both genes, we have identified two *Mansonella* species clusters that correspond to either *M. perstans* or *Mansonella* sp “DEUX”; the same clustering was also seen by Gaillard et al. for *cox1* sequences (Gaillard et al., 2020). We can confirm these two separate clusters also based on the 12S rDNA sequences. Only one cluster for 12S rDNA is shown by Gaillard et al., however, when we reanalyzed Gaillard’s data, we identified two clusters.

Molecular markers based on mitochondrial sequences are a commonly used tool in phylogenetic studies due to the conserved nature among species, and several markers have assisted in recent years to differentiate filarial species and determine the phylogeny of the *Onchocercidae* family (Xie et al., 1994; Lefoulon et al., 2015; Yilmaz et al., 2016; Crainey et al., 2018). The investigated markers 12S rDNA and *cox1* overall support the evolutionary relationships and classification into different clades, as suggested by Lefoulon et al. (2015). Based on multi locus sequence typing they had identified five strongly supported clades, whereby all *Mansonella* species fall within clade ONC5. The affiliation to the different ONC clades is also reflected in our phylogeny based on the complete mitogenome and the phylogeny based on the concatenation of the 562 protein alignments as seen in **Figure 1**.

Our analysis of the *cox1* and 12S rDNA genes additionally showed that our *Mansonella* sp “DEUX” sequences align to *Mansonella* sequences obtained from great apes from the same country (Gabon) and the neighboring country Cameroon (Gaillard et al., 2020), suggesting that *Mansonella* sp “DEUX” is infecting humans as well as NHPs. There are a few *Mansonella* species that have been described to occur in great apes based on morphology, as *M. vanhoofi* (Peel, 1946), *M. gorillae* (van den Berghe and Chardome, 1949), *M. leopoldi* (van den Berghe et al., 1957) *and M. loopensis* (Bain et al., 1995). Most of them have been described in the form of a case report. One ape related species has been described to occasionally infect humans in Gabon – *M. rodhaini* (Richard-Lenoble et al., 1988). There is no sequence information for any of these species available to compare them on a genetic level to our assemblies. However, *M. rodhaini* is described to be a skin dwelling species like *M. streptocerca* and not a blood dwelling species like *M. perstans* or *Mansonella* sp “DEUX”. We also compared our obtained sequences to the few available sequences of the skin dwelling *M. streptocerca*, but they did not map. Therefore, there is no indication that these skin dwelling species are the same as *Mansonella* sp “DEUX”. To determine if one of the morphologically described great ape infecting *Mansonella* species could be *Mansonella* sp “DEUX”, microscopic investigations of mono-infected samples could be done.

We additionally identified *Wolbachia* reads in all three sequenced samples. *Wolbachia* are intracellular bacteria (order *Rickettsiales*) found in a range of arthropod and nematode species (Taylor et al., 2018). In filarial nematodes *Wolbachia* are usually symbionts, beneficial for the host survival and reproduction (Bandi et al., 1999; Hoerauf et al., 1999; Volkmann et al., 2003). Antibiotic treatment of filarial infections targets the *Wolbachia* and are promising alternative treatment options, especially for *M. perstans* infections, where standard filarial treatments are not efficacious (Coulibaly et al., 2009; Raoult, 2010; Batsa Debrah et al., 2019). Currently, *Wolbachia* are taxonomically subdivided into 16 different so-called supergroups. *Wolbachia* of filaria fall in supergroups C, D, and J with the exception of *Wolbachia* of *Mansonella* species that fall in supergroup F. Interestingly, all other *Wolbachia* of supergroup F have arthropod hosts (Covacin and Barker, 2007; Ferri et al., 2011). We could confirm that *Wolbachia* reads from *Mansonella* sp “DEUX” also belong to supergroup F, similar to *Wolbachia* of *M. perstans* and *M. ozzardi* (Casiraghi et al., 2001; Keiser et al., 2008). Whole genomes as well as sequence comparisons of the *Wolbachia* of these two *Mansonella* species (*M. perstans and M. ozzardi*) are described in a preprint (Sinha et al., 2021). *Wolbachia* are usually inherited maternally and differences between *Wolbachia* of different species can give further evidence on their relatedness. The read coverage of our assembled *Wolbachia* genomes was low (<10x) leading to a high fragmentation with many small contigs. However, the finding of *Wolbachia* reads in all three samples, further supports the carriage of *Wolbachia* in *M. perstans* and *Mansonella* sp “DEUX”.

Based on mitogenomic analysis, we found a third *Mansonella* mitogenome that did not cluster with any of the available sequences and might therefore even present another new *Mansonella* species, that we named “*Mansonella cermeli”.* As read coverage was low, we could not construct a separate whole genome, but because of the multicopy nature of mitogenomic DNA in general, we could construct a separate mitogenome. Evidence of this separate genome is high, as the whole mitogenomes could be assembled into a single circular contig. This is the first time that sequences of “*M. cermeli”* are detected, and we do not have a morphologic proof of its existence. Also, we do not know if this is a common species/infection in that region. Further studies must be conducted to analyze if this is another common *Mansonella* species infecting humans. The mitogenomes reported here show that the species diversity within *Mansonella* might even be higher than previously thought.

Overall, the results of our study suggest that *Mansonella* sp “DEUX” represents a new *Mansonella* species. It is most probably not restricted to the human host, but also infects great apes. However, given the high prevalence of this mansonellosis in the population in this area, one can assume that it is also a true human infection. The detection of the additional “*M. cermeli”* mitogenome further illustrates the lack of knowledge about this genus. Fortunately, awareness is increasing and more data are being published, such as a preprint this year describing the entire genomes of *M. perstans* and *M. ozzardi* (Sinha et al., 2023). The authors discuss clinical differences in response to antifungal drugs in the context of molecular differences in selected genes of the different species. These publications together with the sequence data are of great value for further research on *Mansonella*. There are many open questions on *Mansonella* infections not only related to phylogeny and number of infecting species, but also on the burden of disease, adequate treatment and impact on the immune system. However, to properly address these questions, one prerequisite is to be able to identify, name, and differentiate the infecting species.

## Data availability statement

The datasets presented in this study can be found in online repositories. The names of the repository/repositories and accession number(s) can be found below: https://www.ebi.ac.uk/ena, PRJEB57801 and PRJNA942613.

## Author contributions

JH, TS, SO, SA, NC designed the study. LB collected samples. MR, LB, MP performed lab experiments. MR, CG, TS, MM-H, EK, TG, SO, JH analyzed data. TS, NC, AK, SA interpreted results. TG, NC, CK, AK, JH provided resources. MR, CG, JH wrote the first draft. All authors contributed to the article and approved the submitted version.
