# Supplementary material for: Whole genome analysis of two sympatric human Mansonella: Mansonella perstans and Mansonella sp “DEUX”
Source: Front Cell Infect Microbiol. 2023 Apr 14;13:1159814. doi: 10.3389/fcimb.2023.1159814 (PMC10145164; doi:10.3389/fcimb.2023.1159814)
Supplement: Supplementary file 1 [file Table_1.pdf]

Supplementary Material

Whole genome analysis of two sympatric human *Mansonella*: *Mansonella perstans* and *Mansonella* sp "DEUX"

Miriam Rodi[1†], Caspar Gross[2†], Thaisa Lucas Sandri[1,3], Lilith Berner[1], Marina Marcet-Houben[4,5], Ersoy Kocak[6], Michaela Pogoda[2,6], Nicolas Casadei[2,6], Carsten Köhler[1,7], Andrea Kreidenweiss[1,7,8], Selidji Todagbe Agnandji[1,7], Toni Gabaldón[4,5,9,10], Stephan Ossowski[2†], Jana Held[1,7,8†*]

*** Correspondence:**
Jana Held
jana.held@uni-tuebingen.de

Supplementary File 1. De novo assembly information on Kraken and gene annotation.

Kraken. Kraken2 was used to taxonomically classify all generated reads. The pre-built Kraken database composition was as followed:

Bacteria (85.13%, 358273085 bp)

Eukaryota (11.29%, 47508472 bp)

Archaea (2.56%, 10774261 bp)

Viruses (0.98%, 4115713 bp)

Viroids (0.00%, 277 bp)

The database can be downloaded from the following link: https://doi.org/10.5281/zenodo.3259407

Gene annotation.

```
exonerate --model protein2genome --showtargetgff T --subopt F --maxintron 30000 --proteinwordlen 10 --wordjump 3 --percent 95 brugia_malayi_proteins.fasta {input.scaffolds} > {hints.output}
```

```
exonerate2hints.pl --in={hints.output} --source=P --out=hints_protein.gff
```

```
augustus --species=brugia --strand=both --genemodel=complete --gff3=on --protein=1 --extrinsicCfgFile=extrinsic.M.RM.E.W.P.cfg --codingseq=1 --hintsfile=hints_protein.gff --outfile={output.gene} {input.scaffolds}
```

```
getAnnoFasta.pl {output.gene}
```

Supplementary Table 1. In the *de novo* assembly process, so-called unclassified reads were blasted to compare their similarity to other nematode sequences. If one of the 39 nematode species listed below appeared among the first 10 BLAST hits, this scaffold was considered as a fragment from a *Mansonella* spp. genome.

Acanthocheilonema reconditum	*Litomosa westi*	*Dirofilaria repens*	*Onchocerca gibsoni*
Acanthocheilonema viteae	*Litomosoides brasiliensis*	*Dirofilaria tenuis*	*Onchocerca gutturosa*
Brugia malayi	*Litomosoides scotti*	*Dirofilaria ursi*	*Onchocerca lupi*
Brugia pahangi	*Litomosoides sigmodontis*	*Elaeophora abramovi*	*Onchocerca volvulus*
Brugia timori	*Litomosoides wilsoni*	*Elaeophora bohmi*	*Piratuba digiticauda*
Cercopithifilaria johnstoni	*Loa loa*	*Elaeophora elaphi*	*Sarconema eurycerca*
Chandlerella quiscali	*Mansonella ozzardi*	*Elaeophora poeli*	*Waltonella flexicauda*
Dipetalonema reconditum	*Mansonella perstans*	*Elaeophora sagitta*	*Wuchereria bancrofti*
Dipetalonema repens	*Mansonella streptocerca*	*Elaeophora schneideri*	*Wuchereria kalimantani*
Dirofilaria immitis	*Ochoterenella digiticauda*	*Foleyella furcata*	

Supplementary Table 2. List of nematode species added to the analysis, including BUSCO results for all proteomes.

Species name	Download site	Bioproject / Code	Used in phylome	BUSCO complete	BUSCO missing
Acanthocheilonema viteae	NCBI	ASM90053725v1 / PRJEB1697	Yes	85.2%	10.7%
Brugia malayi	NCBI	ASM299v2 / PRJNA10729	Yes	93.7%	3.1%
Brugia pahangi	NCBI	PRJEB497	Yes	91.1%	5.4%
Brugia timori	NCBI	PRJEB4663	No	60.0%	27%
Caenorhabditis elegans	Quest for orthologs 2020	UP000001940	Yes	NA	NA
Litomosoides sigmodontis	NCBI	ASM90053727v1 / PRJEB3075	Yes	88.9%	7.9%
Loa loa	NCBI	PRJNA37757	Yes	98.2%	0.3%
**mperst1**	NCBI	PRJEB57801	Yes	92.8%	5.3%
**mdeux3**	NCBI	PRJEB57801	Yes	94.8%	4.1%
**mdeux2**	NCBI	PRJEB57801	-	94.3%	4.4%
Onchocerca flexuosa	NCBI	PRJNA230512	No	66%	30%
Onchocerca ochengi	NCBI	PRJEB1465	Yes	84.2%	10%
Onchocerca volvulus	Uniprot	UP000024404	Yes	98.2%	1.3%
Thelazia callipaeda	NCBI	PRJEB1205	Yes	89.1%	7.6%
Toxocara canis	NCBI	PRJNA248777	Yes	87.7%	8.7%

Supplementary Table 3. Accession numbers of all 17 nematode mitogenomes used for the generation of the mitogenomic phylogeny.

GenBank ID	Species
NC_016186	*Wuchereria bancrofti*
NC_004298	*Brugia malayi*
CM022469	*Brugia pahangi*
AP017686	*Brugia timori*
KT599912	*Onchocerca volvulus*
MW266120	*Onchocerca lupi*
HQ214004	*Onchocerca flexuosa*
KX181289	*Onchocerca ochengi*
HQ186250	*Loa loa*
MT361687	*Mansonella perstans*
KX822021	*Mansonella ozzardi*
AJ537512	*Dirofilaria immitis*
KX265048	*Dirofilaria repens*
HQ186249	*Acanthocheilonema viteae*
HM773029	*Chandlerella quiscali*
GU138699	*Setaria digitata*
MH937750	*Setaria labiatopapillosa*

Supplementary Table 4. Comparison of genome assemblies of mperst1, mdeux2 and mdeux3 to other filarial whole genomes.

	mperst1	mdeux2	mdeux3	*L. loa*	*B. malayi*	*B. pahangi*	*W. bancrofti*	*O. volvulus*	*O. ochengi*	*D. immitis*	*C. elegans*
**# contigs**	4,659	6,747	3,838	5,761	196	146	704	674	20,243	1,294	6
**Largest contig (kb)**	295	745	919	1,326	24,944	19,997	24,139	28,345	230	779	20,924
**Total length (Mb)**	76.2	80.0	78.5	91.4	87.2	96.4	88.4	96.3	91.7	83.7	100.3
**GC (%)**	30.3	30.3	30.3	31.0	28.4	28.3	29.2	29.2	29.8	27.7	35.4
**N50 (kb)**	45.5	101.8	173.8	174.4	14,214.7	10,892.8	12,368.7	25,485.0	44608	126.5	17,493.8
**N90 (kb)**	10.8	10.3	22.1	18.8	13,467.2	252.6	1918.6	2816.6	1.6	30.6	13,783.8
**auN**	60,258	138,565	232,490	279,459	16,266,090	10,155,925	12,524,268	20,742,403	28,290	172,512	17,038,037
**L50**	471	215	127	130	3	4	3	2	1317	187	3
**L90**	1757	1004	570	690	5	25	10	5	8293	706	6
**# N's per 100 kbp**	4.9	229.8	30.2	4210.9	318.2	0.01	1585.3	3190.9	417.6	0.5	0.0
**Complete BUSCO (%)**	94.7	94.4	96.0	95.4	96.4	95.4	96.4	95.1	92.1	95.7	96.4
**Partial BUSCO (%)**	1.7	2.3	0.7	1.0	1.0	1.3	1.0	1.3	4.3	0.3	0.0
**# predicted rRNA genes**	21 + 6 part	22 + 6 part	22 + 5 part	11 + 1 part	99 + 1 part	156 + 3 part	11 + 2 part	11 + 2 part	5 + 4 part	13 + 8 part	17 + 0 part
**Assembly**	PRJEB57801	PRJEB57801	PRJEB57801	GCF_000183805.2_Loa_loa_V3.1_genomic	GCF_000002995.4_B_malayi-4.0_genomic	GCA_012070555.1_ASM1207055v1_genomic	GCA_005281725.1_ASM528172v1_genomic	GCA_000499405.2_ASM49940v2_genomic	GCA_000950515.2_O_ochengi_Ngaoundere_genomic	GCA_013365355.1_Dim_FR3_v1_genomic	GCA_000002985.3_WBcel235_genomic

Supplementary Table 5. *Wolbachia* reads from the samples mperst1 (wMpe1), mdeux2 (wMde2) and mdeux3 (wMde3) in comparison to published *Wolbachia* sequences from *M. perstans* (wMpe), *M. ozzardi* (wMoz), *Brugia malayi* (wTRS), *Madathamugadia hiepei* (wThie) and *Cimex lectularius* (wCle). NA = not applicable

	***w*Mpe1**	**Mde2**	**Mde3**	**Mpe**	**Moz**	**Mhie**	**Cle**	***w*TRS**
**# contigs (>= 0 bp)**	1746	2061	3562	170	93	208	1	1
**# contigs (>= 1000 bp)**	45	181	401	152	90	198	1	1
**# contigs (>= 5000 bp)**	0	0	1	77	66	72	1	1
**# contigs (>= 10000 bp)**	0	0	0	35	46	26	1	1
**# contigs (>= 25000 bp)**	0	0	0	1	9	0	1	1
**# contigs (>= 50000 bp)**	0	0	0	0	0	0	1	1
**Total length (>= 0 bp)**	852,63	1,127,394	1,838,956	1,058,123	1,073,310	1,025,329	1,250,060	1,080,084
**Total length (>= 1000 bp)**	60,797	237,271	565,576	1,044,843	1,071,154	1,017,416	1,250,060	1,080,084
**Total length (>= 5000 bp)**	0	0	5642	837,505	1,003,057	667,035	1,250,060	1,080,084
**Total length (>= 10000 bp)**	0	0	0	537,097	858,550	347,931	1,250,060	1,080,084
**Total length (>= 25000 bp)**	0	0	0	28,485	288,557	0	1,250,060	1,080,084
**Total length (>= 50000 bp)**	0	0	0	0	0	0	1,250,060	1,080,084
**# contigs**	314	537	916	170	93	208	1	1
**Largest contig (bp)**	3,455	4,757	5,642	28,485	37,481	21,863	1,250,060	1,080,084
**Total length (bp)**	253,074	489,694	932,800	1,058,123	1,073,310	1,025,329	1,250,060	1,080,084
**GC (%)**	32.46	32.83	33.50	35.37	35.64	36.09	36.25	34.18
**N50**	821	989	1,14	10,041	17,225	6,845	1,250,060	1,080,084
**Accession Number**	NA	NA	NA	JACZHU01.1	JADAKK01.1	AS1336685	AP013028.1	NC006833.1
**Wolbachia host**	Mansonella perstans	Mansonella sp „DEUX"	Mansonella sp „DEUX"	Mansonella perstans	Mansonella ozzardi	Madathamugadia hiepei	Cimex lectularius	Brugia malayi
**Wolbachia supergroup**	NA	NA	NA	F	F	F	F	D

Supplementary Table 6. Values of amino acid and nucleotide identity between different pairs of species. Values are calculated as the average between 100 pairs of BRH and their standard deviation.

Species 1	Species 2	Average AA identity	Average nucleotide identity
B. timori	*B. pahangi*	96.9% ± 3.7%	98.0% ± 1.9%
B. timori	*B. malayi*	97.2% ± 5.6%	98.5% ± 3.8%
B. pahangi	*B. malayi*	97.2% ± 3.8%	98.4% ± 1.3%
**mdeux3**	**mdeux2**	99.5% ± 3.3%	99.8% ± 1.4%
**mdeux3**	**mperst1**	98.4% ± 2.8%	99.1% ± 1.3%
**mdeux2**	**mperst1**	97.7% ± 4.3%	98.7% ± 2.6%
O. volvulus	*O. flexuosa*	92.0% ± 6.6%	94.3% ± 2.7%
O. volvulus	*O. ochengi*	98.3% ± 3.7%	99.2% ± 2.0%
O. flexuosa	*O. ochengi*	91.3% ± 8.3%	93.8% ± 4.2%
